# Phylogenetic analyses of Eurasian lynx (*Lynx lynx* Linnaeus, 1758) including new mitochondrial DNA sequences from Iran

**DOI:** 10.1038/s41598-022-07369-z

**Published:** 2022-02-28

**Authors:** Farshad Behzadi, Mansoureh Malekian, Davoud Fadakar, Mohammad Ali Adibi, Eva V. Bärmann

**Affiliations:** 1 Department of Natural Resources, Isfahan University of Technology, 84156-83111 Isfahan, Iran; 2 Department of Environmental Science, Science and Research Branch, Islamic Azad University, Tehran, Iran; 3 Zoological Research Museum Alexander Koenig, Adenauerallee 160, 53113 Bonn, Germany

**Keywords:** Evolutionary genetics, Population genetics, Speciation, Taxonomy, Phylogenetics

## Abstract

The Eurasian lynx (*Lynx lynx*) is one of the widespread felids in Eurasia; however, relatively little is known about the Asian subspecies, and especially the Iranian populations, which comprise the most southwestern part of its range. The current study aimed to assess the phylogenetic status of Iranian populations relative to other populations of Eurasia, by sequencing a 613 bp fragment of the mitochondrial control region. In total, 44 haplotypes were recorded from 83 sequences throughout Eurasia, two of which were found in Iran. The haplotype (H1) is dominant in all Iranian lynx populations and identical to specimens from SW Russia and central China. The second haplotype (H2) is unique and was recorded only from Ghazvin Province in the central Alborz Mountains. Both haplotypes occur in Ghazvin Province. The phylogenetic tree and a median-joining network identified four clades (i.e., East, West 1, West 2, and South). These results are congruent with previous studies and suggest that Eurasian lynx was restricted to the southern part of its range during the glacial maxima and expanded from there to East Asia and to Europe during several independent re-colonization events. The Caucasus region most like plays an important role as a refugium during glacial cycles.

## Introduction

Eurasian lynx (*Lynx lynx* Linnaeus, 1758) is one of the widespread felids in Eurasia, distributed from western Norway to east of the Kamchatka Peninsula in the Russian Far East^1–3^. The species has been classified as ‘Least Concern’ in the IUCN red list due to its wide range and stable populations in Europe^4^; however, status and trend vary greatly across its range^1,4^.

Based on the morphological differences, 11 subspecies have been recognized for the Eurasian lynx^5^. However, only six subspecies are proposed by the IUCN Cat Specialist Group, including northern lynx (*L. l. lynx*), Balkan lynx (*L. l. balcanicus*), Carpathian lynx (*L. l. carpathicus*), Caucasian lynx (*L. l. dinniki*), Turkestan lynx (*L. l. isabellinus*), and Siberian lynx (*L. l. wrangeli*)^6^. Iranian populations of the Eurasian lynx are assigned to *L. l. dinniki*^7^, distributed in Caucasus Mountains, Asia Minor, Iraq, and Iran^6^.

Caucasian lynx is broadly, but patchily, distributed across Iran in a variety of habitats, including the Hyrcanian forests along Alborz Mountain Range, Zagros oak forests from the north-west towards the south-west, and the semi-arid highland steppes of southern Alborz Mountain Range as far as north-eastern Iran^8,9^. The subspecies is currently listed as “protected” under Iranian environmental conservation laws and regulations. Habitat destruction and fragmentation are suggested to be the main threats, which occur particularly through deforestation (expansion of agricultural and residential areas) in north and northwestern Iran^8,10^.

The evolutionary history and population genetics of the Eurasian lynx have received much attention in Europe and Siberia^11–17^. Three phylogenetic clades have been described which overlap partially, suggesting a common evolutionary history of the lynx populations until recent times^15,17^. Gugolz et al.^17^ suggested a postglacial recolonization scenario for the Eurasian lynx in Europe after the Last Glacial Maximum. In another large scale phylogenetic study, using mitochondrial control region and cytochrome *b* sequences as well as microsatellites, Rueness et al.^15^ found that the most ancient lynx lineage exists in central Asia, proposing that Eurasian lynx populations originated in central Asia and expanded to north-western Siberia and Scandinavia after the Last Glacial Maximum. Lucena-Perez et al.^16^ studied the genomic patterns of five out of the six subspecies; however, no samples of *L. l. dinniki* were included. Thus, the phylogenetic status of this subspecies remained unclear.

Although a large portion of the lynx’s distribution occurs in Asia, insufficient sampling left a large gap of knowledge in the natural history and phylogenetic status of this species, particularly in southwestern Asia including Iran. Current knowledge of the Eurasian lynx in Iran is limited to studies which addressed the distribution of the species^8,10^. Phylogenetic status and genetic diversity of the subspecies are yet to be investigated. Understanding of the evolutionary history and molecular genetic variation of the subspecies is necessary to develop management and conservation plans. The current study aimed to assess the phylogenetic relationship of Iranian lynx populations in relation to other populations using mitochondrial DNA (mtDNA) control region sequence variation. Control region is a non-coding and fast evolving sequence of the mitochondrial genome^18,19^ which is a standard marker in population genetic analysis of mammals^20–22^ and has been used in phylogenetic studies of the Eurasian lynx across Eurasia^11–13,15,17^.

## Results

**Figure 1:**
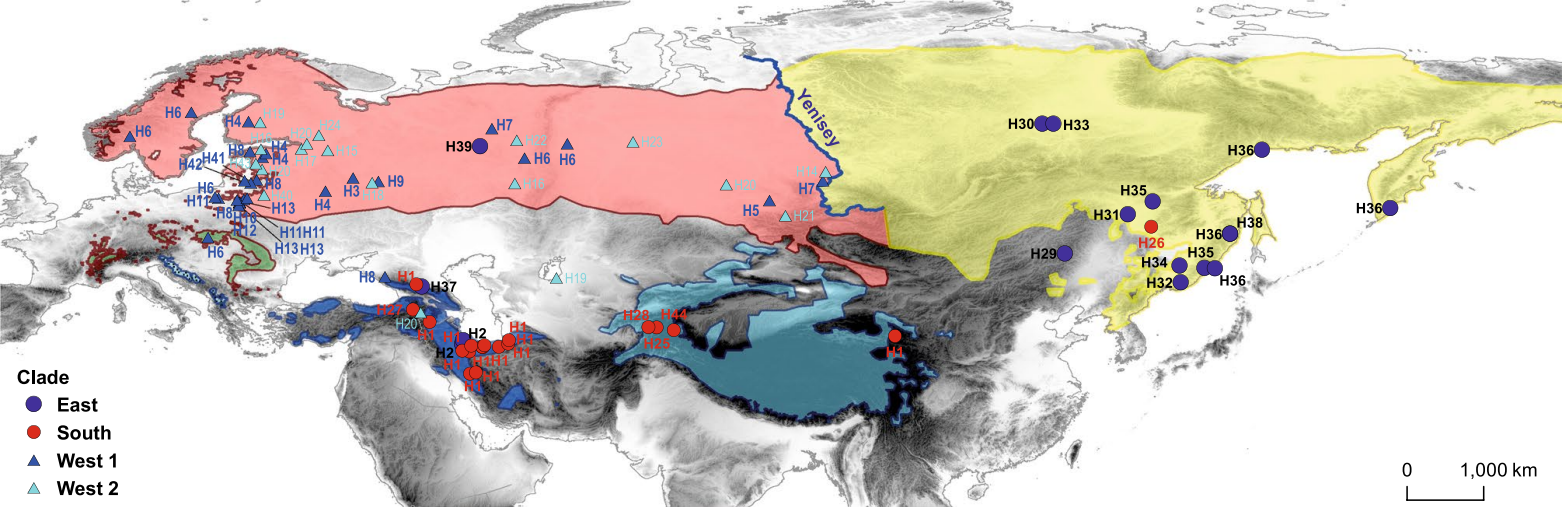
Distribution of subspecies of *L. lynx* based on the morphological data and CR sequences. Yellow (*L. l. wrangeli*), pink (*L. l. lynx*), green (*L. l. carpathicus*), light blue (*L. l. isabellinus*), blue (*L. l. dinniki*), and doted light blue (*L. l. balcanicus*) polygons correspond to the distribution based on the six suggested subspecies by the IUCN Cat Specialist Group^6^ which are modified from distribution map of *L. lynx*^4^, circle and triangle represent samples sequenced for CR and showed four recognized clades. Different shades of gray represent different altitudes.

In total, the control region (CR) fragment of 15 samples of Iranian lynx was successfully amplified. All new mtDNA sequences were submitted to GenBank (accession numbers OM743776-OM743790). These sequences were aligned with 68 published Eurasian lynx D-loop sequences, retrieved from GenBank. The available mtDNA sequences were slightly trimmed to match our sequence length, therefore we added only 38 instead of 48 haplotypes detected by Rueness et al.^15^, plus five haplotypes from other sources^12,13^ (Fig. 1). A complete list of the mtDNA sequences and corresponding haplotypes used in the present study are given in Supplementary Table S1, supporting information.

### Haplotype network

**Figure 2:**
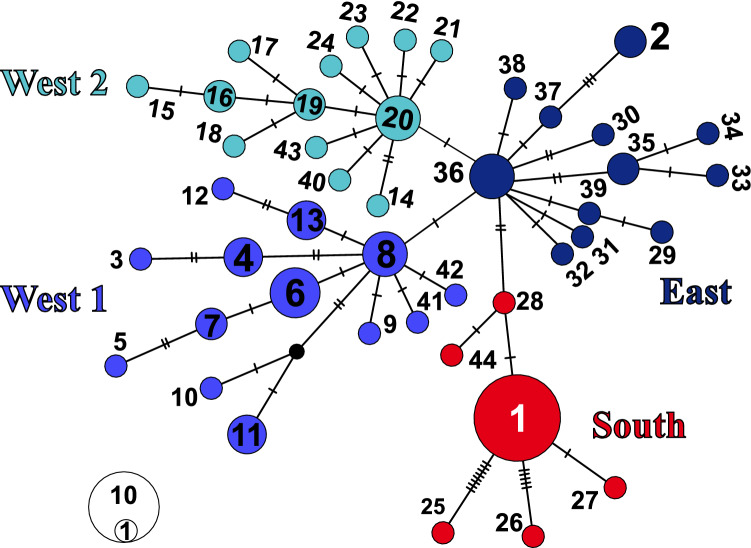
Median-joining network based on CR gene depicting the relationships between the main four groups described for Eurasian lynx including East (purple), South (red), West 1 (blue), and West 2 (turquoise). The colors correspond to the respective clades in the phylogenetic tree. Additional details of haplotypes and accession numbers are provided in Supplementary Table S1, supporting information. Black small dots represent missing haplotype, circle sizes are proportional to haplotype frequencies, and numbers are haplotype numbers. H1 and H2 were found in Iran.

Based on 613 base pairs (bp) of CR from 83 mtDNA sequences, 44 unique haplotypes (including one new haplotype from Iran) were defined by 61 variable sites. The overall level of genetic variability in Iran was very low with only two haplotypes (H1 and H2) found. A median-joining (MJ) network illustrating the relationship between Eurasian lynx haplotypes is depicted in Fig. 2. Four haplogroups can be differentiated: South, West 1, West 2, and East.

Haplotype H1 was the most common haplotype among Iranian samples, occurring in 86% of Iranian samples (13 individuals) and was identical with EU818858 from Southwest Russia (approximate latitude and longitude: 43.2, 44.35) and EU818861 central China (approximate latitude and longitude: 37.3, 100). H1 belongs to haplogroup South.

The second haplotype H2 was unique, occurring only in two specimens (GHZV1 and GHZV2) from Ghazvin Province in the south of the Caspian Sea (approximate latitude and longitude: 35.36, 49.14). H2 was connected to H37 in the Caucasus region, and formed part of haplogroup East (Fig. 2). In contrast, another sample from Ghazvin (GHZV3) was found to possess H1. Therefore, both recorded haplotypes for Iran occur in Ghazvin Province in central Alborz Mountain Range.

### Phylogenetic analysis

In the preliminary analyses, *Lynx canadensis* (AY319505) rooted the tree within the South clade between (H1, H25, H26, H27) and the remaining haplotypes, *Lynx pardinus* (KF561247) between H26 and the remaining haplotypes, while *Lynx rufus* (NC014456), *Caracal caracal* (KP202272), and *Leopardus jacobita* (FJ960826) rooted the tree within the East clade (large polytomy). Including all outgroup representatives lead to a badly resolved tree with a huge polytomy (within the South clade) at the base of the ingroup.

**Figure 3:**
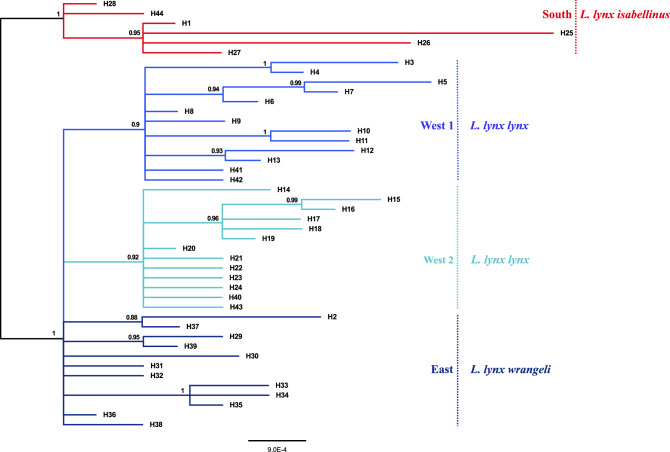
Phylogeny of Eurasian lynx based on partial mitochondrial control region sequences. Consensus tree from Bayesian analysis of 44 haplotypes using the HKY + G substitution model. Numbers at branches indicate support from posterior probability. The colors of the lines correspond to groups in the haplotype network: red = South, blue = West 1, turquoise = West 2, purple = East.

The phylogeny from the ingroup-only analysis showed two separate groups, a southern group (posterior probability (PP) = 1, Fig. 3) with samples from southern Eurasia (South clade), and a northern group comprising samples from northern Eurasian localities. The northern group was further subdivided into one paraphyletic Asian group (East clade) and two monophyletic European groups (West 1 (PP = 0.9) and West 2 (PP = 0.92), Fig. 3). This pattern corresponds with the haplotype network (Fig. 2). Accordingly, the new mtDNA sequences from Iran belonged to both, the southern and the northern groups. In the analysis with the trimmed alignment (326 bp) only one split was well supported (PP = 1, Supplementary Fig. S1): the split between the southern group and the northern group. The haplotype from the Balkan region grouped with our clade South in the analysis with the trimmed alignment (including only the 326 bp also present in Lucena-Perez et al.^16^, Supplementary Fig. S1). The four other haplotypes of Lucena-Perez et al. were included in the northern group, but no further structure within this northern group was recovered.

In the time-calibrated CR mitochondrial phylogeny, the split between Eurasian lynx and other lynx species (*L. rufus*, *L. pardinus*, and *L. canadensis*) is well supported (PP = 1, Supplementary Fig. S2). The split of European lynx and the sister species Canadian lynx was estimated to have occurred around 45.6 kya (95% confidence interval [CI]: 30.8–62.4 kya). Within Eurasian lynx, the northern group (West 1 + West 2 + East) was found monophyletic and well supported (PP = 0.97), with the corresponding split from the southern group dated to around 15.7 kya (95% CI: 9.7–22.4 kya). However, each of the four subclades found in the ingroup-only analysis and the haplotype network (South, West 1, West 2, and East) did not receive support from posterior probabilities (PP < 0.7 in all four groups), so reporting on the divergence times between these groups seems inadequate considering the very limited amount of data for this analysis.

### Population differentiation and structure

In order to obtain an overview of the CR genetic diversity of *L. lynx*, basic molecular diversity indices were determined for the species and for three proposed subspecies that correspond to the geographic groups of southern, eastern, and western samples (Table 1). The highest level of genetic diversity was found in the nominate subspecies *L. l. lynx* (corresponding to clades West 1 and West 2) with *h*: 0.848 ± 0.027 and *π*: 0.00484 ± 0.00031. The haplotype diversity of *L. l. wrangeli* (East clade, *h*: 0.574 ± 0.089) is higher than the *L. l. isabellinus* **(**South clade, *h*: 0.411 ± 0.131; *π*: 0.00251 ± 0.00122), while its nucleotide diversity is lower (*π*: 0.00229 ± 0.00051).

**Table 1:** CR mtDNA genetic diversity revealed for proposed subspecies of *L. lynx*.

	n	S	H	*h* ± std. dev	*π* ± std. dev	*k*
*Lynx lynx* (all sequences)	172	61	44	0.906 ± 0.012	0.00534 ± 0.00033	3.27173
*L. l. isabellinus* (South clade)	22	16	6	0.411 ± 0.131	0.00251 ± 0.00122	1.5368
*L. l. wrangeli* (East clade)	43	16	12	0.574 ± 0.089	0.00229 ± 0.00051	1.40642
*L. l. lynx* (West 1 + West 2 clades)	107	32	26	0.848 ± 0.027	0.00484 ± 0.00031	2.96632

*n* number of individuals, *S* number of segregating sites, *H* number of haplotypes, *h* haplotype diversity, *π* nucleotide diversity, *k* mean number of pairwise differences.

**Figure 4:**
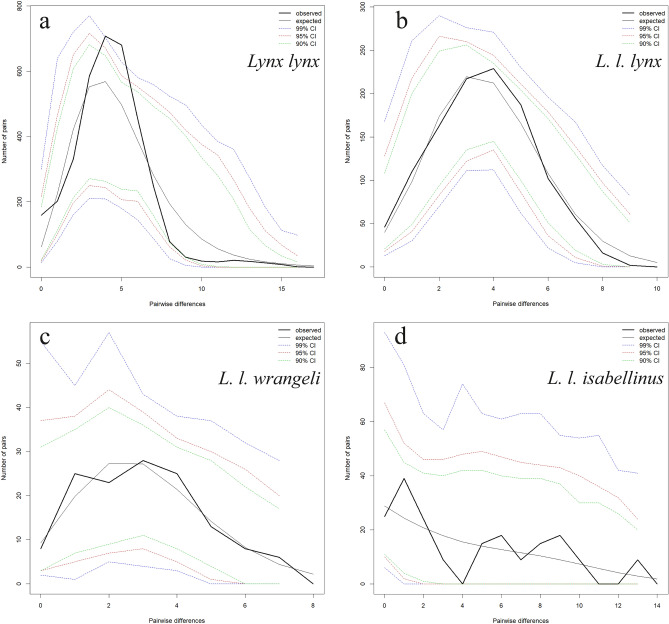
Mismatch distributions of pairwise differences of CR haplotypes for the *L. l. lynx* (West 1 clade and West 2 clade) from northern Eurasia in the west of Yenisey river, *L. l. wrangeli* (East clade) from northern Eurasia in the east of Yenisey river, *L. l. isabellinus* (South clade) from southern Eurasia, and for the whole species (*L. lynx*). Depicted are observed (solid black lines) and expected (solid gray lines) frequencies obtained under a model allowing for demographic expansion.

Mismatch distributions were calculated separately for the total group that includes all sequences (*Lynx lynx*), as well as for *L. l. lynx*, *L. l. wrangeli*, and for *L. l. isabellinus* (Fig. 4). The mismatch distributions for the total group (Fig. 4a) and *L. l. lynx* (Fig. 4b) were unimodal and fully consistent with population expansion, but for *L. l. wrangeli* (Fig. 4c) and *L. l. isabellinus* (Fig. 4d) the graphs showed a pattern that is interpreted to represent demographic equilibrium. A similar picture is found using R_2_ statistic (Table 2), which was significant, and therefore supporting population expansion, for *Lynx lynx* (the total group, p < 0.01) and *L. l. lynx* (p < 0.05), but not for *L. l. wrangeli* and *L. l. isabellinus****.*** Fu’s F_s_ values were highly significant (p < 0.01) for the *L. l. lynx*, and all sequences, but it is not significant for the *L. l. isabellinus* and *L. l. wrangeli*.

**Table 2:** Tests for population expansion for proposed subspecies of *L. lynx* using R_2_^48^ and Fu’s F_s_^49^.

	R_2_	Fu’s F_s_
*Lynx lynx* (all sequences)	0.0263**	− 33.099**
*L. l. isabellinus* (South clade)	0.1199 (n.s.)	− 0.848 (n.s.)
*L. l. wrangeli* (East clade)	0.0463 (n.s.)	− 6.4031 (n.s.)
*L. l. lynx* (West 1 + West 2 clades)	0.0432*	− 12.515**

*n.s.* not significant.

*Significant (p < 0.05).

**Highly significant (p < 0.01).

**Figure 5:**
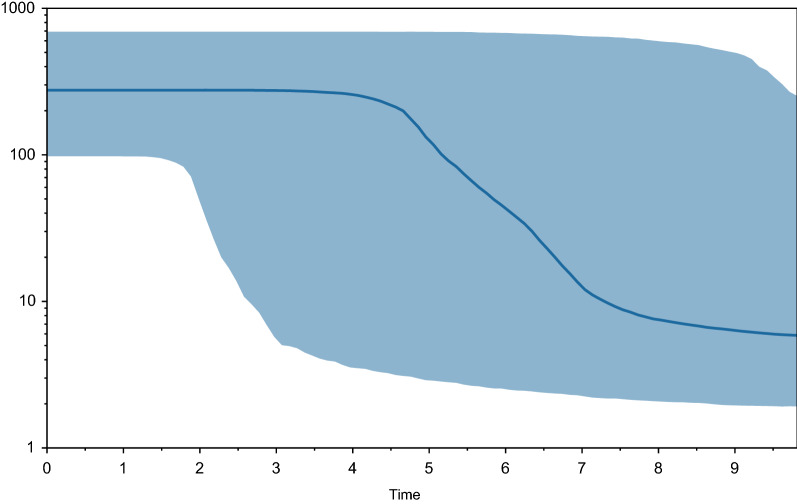
Bayesian skyline plot of mitochondrial DNA control region haplotypes of Eurasian lynx. The x-axis represents time in thousand years ago (kya) and the y-axis shows effective population size of females (Ne) multiplied by generation time (T). The solid line shows the median effective population size (NeT) over time to the present, and the blue areas represent the 95% highest posterior density (HPD).

Bayesian skyline plot (BSP) demonstrated apparent evidence of recent increase in the effective population size in last 10 kya within the Holocene (Fig. 5). Therefore, *L. lynx* was found under expansion and demographically increase over the last 10 kya.

## Discussion

### Population structure and comparison with previous analyses

The distribution of the lynx in Asia is described as continuous, with demographically healthy and well-connected populations^15^; however, the information in most areas is scattered or completely lacking^16^. Rueness et al.^15^ identified three main clades for *Lynx lynx* (western, eastern, and southern) based on the mtDNA, with an east–west gradient, which do not appear to correspond to putative subspecies^6^. Rueness et al.^15^ proposed the southern clade of *Lynx lynx,* inhabiting the Himalayas, and (among others) the Caucasus and other Asian regions, to be the ancestral clade, as it had the highest level of haplotype diversity, was the most diverse clade in terms of private alleles, and formed the sister group to the other two groups in their phylogenetic analysis. However, a robust conclusion on the phylogeographical relationships among populations was hampered due to the low resolution imposed by the few microsatellites and the small mitochondrial region used^16^.

In the genomic study by Lucena-Perez et al.^16^, analysing 80 samples, the authors found only 89 segregating sites arranged in 24 haplotypes within the whole mitochondrial genome of European lynx from a geographical range spanning from Norway to Primorsky Krai in Russia. It has to be noted though that their analysis excluded large parts of the mitochondrial control region due to problems with assembling the sequencing reads in this highly variable region. They arranged their recovered haplotypes in five haplogroups. The Balkan haplotype is the only member of haplogroup 1 which might be the sister-group to the other haplotypes. These are arranged in two western (haplogroup 2 and 3) and two eastern (haplogroup 4 and 5) groups, with haplogroup 3 forming the sister-group to haplogroups 4 + 5 in the mitochondrial phylogeny.

Our results expand on previous data sets of control region sequences^11–13,15,23^, but consider each of the haplotypes instead of haplotypes based on informative sites only, as done by Rueness et al.^15^. We found 61 segregating sites within 631 bp of the mitochondrial control region, identifying 44 haplotypes. These form four clades in the phylogenetic analysis, i.e., South, East (paraphyletic), West 1 and West 2 (Fig. 2). As in Rueness et al.^15^, the South clade might be the sister group to the other groups, and this clade contains the common haplotype found in Iran.

Although we were not able to include the control region sequences by Lucena-Perez et al.^16^ in the final analysis, we suggest that their haplogroups correspond to our haplogroups/clades as follows: (1) our clade West 1 is equivalent to haplogroup 2, i.e., the first western clade to branch off in the phylogeny, as they both include samples from the Carpathians that are not present in the other western clade. Nonetheless, our clade West 1 has a much wider distribution, including regions that were sampled for haplogroup 2 only by Lucena-Perez et al.^16^, which might be caused by including museum sample sequences by Rueness et al.^15^ from populations that are now extinct or severely decimated. In Rueness et al.^15^, the corresponding clade is their clade West. (2) Our clade West 2 is equivalent to haplogroup 3, the other western clade that forms the sister-group to the Asiatic haplogroups in Lucena-Perez et al.^16^. Again, our clade West 2 has a much wider distribution, overlapping widely with clade West 1 from eastern Poland to the Yenisey river. (3) Our paraphyletic clade East corresponds to haplogroups 4 + 5 from Lucena-Perez et al.^16^, but we did not find any differentiation within this group, probably due to the much smaller mtDNA sequence length that we used. In Rueness et al.^15^, their clade Northeast corresponds to our clades West 2 + East. (4) Our clade South corresponds to haplogroup 1 by Lucena-Perez et al.^16^, as their haplotype from the Balkan region grouped with our clade South in the analysis with the trimmed alignment (including only the 326 bp also present in Lucena-Perez et al.^16^, Supplementary Fig. S1). Clade South from Rueness et al.^15^ corresponds to our clade South.

### Taxonomy

Although six subspecies of Eurasian lynx were suggested based on the morphological traits such as fur color, body size, and degree of spottiness^6^, our genetic data is not completely corresponding to these putative subspecies. However, three subspecies might have a genetic basis considering the mitochondrial genome. The Siberian lynx (*L. lynx wrangeli*) corresponds to our paraphyletic clade East, and the combined Asian populations by Lucena-Perez et al.^16^. The nominate northern lynx (*L. lynx lynx*) includes two mitochondrial clades, i.e., clade West 1 and West 2 in both our analysis and the study by Lucena-Perez et al.^16^. The third putative subspecies is distributed in southern localities including populations in the Himalaya region, Tibet, Iran, Iraq, Turkey, and the Caucasus region. It corresponds with our clade South. Two subspecies have been described from these regions, i.e., *L. lynx isabellinus* (Blyth, 1847) with type locality in Tibet, and *L. lynx dinniki* (Satunin, 1915) with type locality in the Caucasus^24^. *L. lynx isabellinus* takes priority over *L. lynx dinniki*, so we propose to use this name for the whole southern subspecies. Unfortunately, there is no large-scale study including these southern localities, and the Balkan and Carpathian populations as well, so their genetic relationship remains speculative. Gugolz et al.^17^ found that *L. l. balcanicus* from the Balkan population and *L. l. dinniki* (here synonymized with *L. l. isabellinus*) from Caucasus share one haplotype and are perhaps con-subspecific^6^, which was also supported by our analysis using the trimmed alignment (326 bp of the control region). During glacial cycles, a land bridge existed between Asia Minor and the Balkans^25^, so it is possible that the Balkan lynx was connected to *L. l. isabellinus*, forming its westernmost population. The only mitochondrial haplotype from the Carpathian region^15^ belongs to clade West 1, and also in Lucena-Perez et al.^16^ the Carpathian population belongs to haplogroup 2, so we consider it to belong to the northern subspecies *L. lynx lynx*. In any case, the genetic distinction between the subspecies is small and implies that morphological traits such as size, fur colour, and texture are, to a large degree, environmentally plastic. Considerable variation in phenotypic traits and division in subspecies, which was not reflected in genetic structuring, has been reported in another lynx species (*Lynx rufus*^26^).

### Population differentiation and taxonomic implications

When looking at population differentiation measures according to the proposed subspecies, the values for genetic diversity of *L. l. lynx* (*h*: 0.848 ± 0.027, π: 0.00484 ± 0.00031) is higher than the *L. lynx wrangeli* (*h*: 0.574 ± 0.089, π: 0.00229 ± 0.00051) and *L. l. isabellinus* (*h*: 0.411 ± 0.131, π: 0.00251 ± 0.00122). It is worth to note that many more sequences are available for *L. l. lynx* than for the other subspecies. Especially for *L. l. isabellinus*, a large part of the range, i.e., the Balkan region, have not been sampled for this study, so the population differentiation could be underestimated for this subspecies.

### Population expansion

The mismatch distribution analysis clearly supports a demographic expansion for the whole *L. lynx* species and *L. l. lynx* subspecies (Fig. 4). For *L. l. wrangeli* the curve is relatively flat with two marked peaks, while multimodal pattern is obvious for *L. l. isabellinus*.

Statistical tests support this interpretation (Table 2): the species as a whole shows clear signs of range expansion in all tests, and this expansion can mostly be attributed to the nominate subspecies (*L. l. lynx*), while the statistics were not significant for *L. l. wrangeli*. Both tests, R_2_ and Fu’s F_s_, did not find evidence for range expansion in *L. l. isabellinus*, so it seems that this southern subspecies has not significantly increased its range compared to the historic distribution and still occurs in the geographic area where it originated in southern Eurasia. This agrees with the assumption that Eurasian lynx was restricted to the southern populations and from there expanded northwards as the habitats became available after the glaciation ended.

The Bayesian skyline Plot also shows population expansion that happened mostly between 8 and 4 thousand years ago, which is after the separation of the southern group (*L. l. isabellinus*) and the northern group (*L. l. lynx* and *L. l. wrangeli*).

### Population history

A previous study on the demographic history of Eurasian lynx concluded that the entire lynx population was very homogenous and large until about 600,000 years ago and started to decline more steeply until 200,000 years ago^16^. Around 100,000 years ago the lynx populations started to diverge genetically, which likely corresponds with several cycles of glaciation that periodically decreased the lynx habitat to small refugia^15^.

Based on the mitogenomic divergence of Eurasian lynx^16^, separation of haplogroup 1, currently restricted to the Balkan population, is the oldest split (around 96.5 kya). This split might also include the southern subspecies from the Himalayas to the Caucasus region and Turkey that was postulated to be the oldest lineage by Rueness et al.^15^. In our study, the oldest split (15.7 kyr) is between the South clade (not including the Balkan region) and the northern clades (West 1, West 2 and East), which is a lot younger, but we urge not to put too much into this as we only analysed a short fragment of mitochondrial DNA.

The next split is the separation of haplogroup 2 (West 1 in our study) from the Carpathian Mountains and the Baltic states populations (around 47.4 kya), followed by the split of haplogroup 3 (West 2 in our study) including other European populations from northern and eastern Europe at around 28.6 kya^16^. It is therefore very likely that the northern subspecies *L. lynx lynx* originated from at least two postglacial colonization events where individuals from southern refugia expanded northwards as the ice retreated. One of these refugia for members of the northern subspecies that originated from the first expansion might be the Carpathian region^12,17,27–29^ with haplotypes belonging only to clade West 1. The occurrence of other southern refugia in Europe is also supported by a number of fossil records from the late Pleistocene^30^.

The Asian haplogroups (East clade in our study) diversified around 17 kya, coinciding with internal diversification within haplogroup 2 (i.e., clade West 1 in our study)^16^. At that time, the split between the northern subspecies *L. lynx lynx* and the Siberian subspecies *L. lynx wrangeli* was complete^16^. It seems that the Yenisey river forms the barrier between *L. l. lynx* and *L. l. wrangeli*, although it has been doubted that it could act as a strong barrier for such a large and mobile carnivore^31^. Only one instance of an Eastern haplotype (H39) found west of the Yenisey river (Fig. 1) was recorded, so there seems to be some possibility for migration across the subspecies boundaries, as also found in Lucena-Perez et al.^16^ between haplogroup 3 in the Urals and the Asian haplogroups.

### The Caucasus region

One of the most interesting and diverse regions in terms of mitochondrial diversity is the Caucasus. It harbors mitochondrial haplotypes from all four haplogroups, i.e., H1 (the most common haplotype found in Iran and also found in the Himalaya region), H8 (the central haplotype of clade West 1), H20 (the central haplotype of clade West 2), and H37 from the East clade. This region is a well-known biodiversity hotspot and the Caucasus forest refugium is the largest throughout the Western Asian/near Eastern region (e.g. Tarkhnishvili et al.^32^). It is very likely that the one of the northwards expansions of the northern group, i.e., clades West 1, West 2, and East, started in this region.

## Conclusion

The present study provides new insights into the genetic diversity of Iranian lynx populations, and their relationships with other Eurasian lynx. Our research demonstrates the importance of southwestern Asia to address questions regarding genetic relationships among the Asian and European lynx populations. The central haplotypes of two clades (H8 for West 1 and H20 for West 2) that colonized Europe exist in the Caucasus beside H1 (most frequent haplotype of South clade) and H37 (East clade); therefore, the Caucasus region was an important refugium for the Eurasian lynx during the glacial maxima, and one of sources for subsequent colonization of northern habitats after the Last Glacial Maximum. The population expansion signatures for the Eurasian lynx, star-like patterns in phylogenetic networks, and population expansion times support recent and quick colonization of northern Eurasia, and appear to reflect responses to postglacial climate warming. Future genetic studies with larger sample size from Iran and neighboring countries such as Iraq and Afghanistan are required to further clarify the evolutionary history of the Eurasian lynx and the taxonomic status of the putative subspecies *L. l. isabellinus*, synonymised here with (and taking priority over) *L. l. dinniki*, and *L. l. balcanicus*.

## Material and methods

### Sampling

The elusive nature of the lynx, which is a highly mobile solitary predator, makes field observation and sampling very difficult. Previous field surveys conducted extensive and intensive camera trapping within several protected areas across the country, but were unsuccessful in capturing any photograph of the species (e.g., Hamidi et al.^33^ and Moqanaki et al.^34^). In addition to low detectability, the population size of the lynx in Iran is unknown. Therefore, optimizing a sampling protocol is a major challenge for Iranian researchers. Some previous research relied on museum specimens, for example, Rueness et al.^15^ used about 150 museum specimens collected between 1844 and 2002.

We collected samples across the species range in Iran, including north, northwest, and west, with the permission of and in accordance with the national regulations of the Department of Environment. A total of 15 samples were collected, including fresh muscle or skin tissues obtained from dead animals (e.g. road killed individuals) (Fig. 1, Supplementary Table S1). Samples were preserved in 96% ethanol and stored at − 20 °C, prior to DNA extraction.

### DNA extraction, amplification and sequencing

Whole genomic DNA was extracted from tissue samples using AccuPrep genomic DNA extraction tissue kit (Bioneer Co., South Korea) following the manufacture’s instructions. Polymerase chain reaction was performed for amplification of a 613 bp fragment of the control region using, mtU (5ʹ-CTTTGGTCTTGTAAACCAAAAAA-3ʹ) and R3 (5ʹ-TAAGAACCAGATGCCAGGTA-3ʹ) primers^35^. Amplifications were performed in 25-μl volumes, containing 1 unit of Euro Taq DNA polymerase, 10 μM Tris–HCl, 30 μM KCl, 1.5 mM MgCl_2_, 250 μM of each dNTP and 2 pmol primers (Bioneer, South Korea). The thermocycling was performed in a *SensoQuest* thermocycler using an initial denaturing at 95 °C for 4 min followed by 40 cycles of 30 s at 94 °C, 35 s at 55 °C, and 50 s at 72 °C, and a final extension at 72 °C for 5 min. Sanger sequencing was performed using the BigDye Terminator Cycle Sequencing kit v.3.1 (Applied BioSystems) and electrophoresis of the purified sequencing product was carried out on an ABI PRISM 3730xl automatic sequencer.

### Alignment and haplotype network

Sequences were edited with SeqScape v.2.6 (Applied Biosystems), and aligned with 68 previously published mtDNA sequences from GenBank using the Clustal W algorithm^36^ implemented in Mega v.5^37^, and checked visually. Unfortunately, the mitochondrial genomes generated by Lucena-Perez et al.^16^ could not be included in the final analysis, as their control region sequences only matched our (and other previously published) lynx sequences for the first 211 bp and the last 115 bp. However, we made a trimmed alignment for a Bayesian analysis (same settings as in the final analysis, see below) and restricted the sequences to the parts that overlapped with the sequences from Lucena-Perez et al.^16^.

DnaSP v.5^38^ was used to find the number of haplotypes and the distribution map of haplotypes was made in QGIS v.3.10. A MJ network was constructed using PopART v.1.7^39^ with the default settings.

### Phylogenetic analysis

For the phylogenetic analysis, we included only one representative of each haplotype. HKY + G was selected as the best model of nucleotide substitution based on the Akaike information criterion (AIC) using jModelTest v.0.1.1^40^. Bayesian phylogenetic tree was constructed in MrBayes v.3.2.7a^41^, using two independent runs of four Markov Chain Monte Carlo (MCMC), ran simultaneously for 10,000,000 generations and sampling every 1,000 generations. The first 25% of the sampled trees and estimated parameters were discarded as burn-in. Convergence of the model parameters was monitored using the program Tracer v.1.7.1^42^. The consensus phylogenetic tree was then edited in FigTree v.1.4.4 (http://tree.bio.ed.ac.uk/software/figtree/). MtDNA is more divergent (having more parsimony informative sites) compared with yDNA, nDNA, and xDNA in the felid species, but its homoplasy index is higher; therefore, mtDNA is not suitable to separate felid species^43^. In preliminary phylogenetic analyses, we used different outgroup representatives for rooting the phylogeny of Eurasian lynx, but different species (e.g., *Lynx canadensis* (AY319505), *Lynx rufus* (NC014456), *Lynx pardinus* (KF561247), *Caracal caracal* (KP202272), and *Leopardus jacobita* (FJ960826)) rooted the tree at different branches and we could not decide a priori which of these rootings was the most appropriate. Although it can be regarded as certain that the Canadian lynx is the sister species to the Eurasian lynx, the rooting by this species might still be artificial due to long branch attraction in this fast-evolving marker. We therefore decided to analyse only the ingroup in the phylogenetic analysis.

The BEAST v.2.6.6 program package^44^ was employed to co-estimate topology and divergence times using a strict clock model. This analysis included one representative each for all other species in the genus *Lynx*. We used the BEAUti program to set up the MCMC run using the following parameters: the best corresponding model (HKY + G) as indicated by jModelTest v.0.1.1^40^, a strict clock, and a calibrated Yule model as the tree prior. The MCMC analyses were run for 50 million generations with sampling each 5,000 generations. We used calibration of Rueness et al.^15^ based on age calibration on priors from Johnson et al.^43^. The log-normal distribution of the root prior (i.e. the split between *L. rufus* (NC014456) and the other lynx species, including Eurasian lynx haplotypes, *L. canadensis* (AY319505), and *L. pardinus* (KF561247)) was set (M = 0.4762 and S = 0.2445) with mean of 1.61 (95% CI 1.06–2.60) Myr and M = 0.1655 and S = 0.2641 of the log-normal distribution were set approximately for the internal node prior (i.e. the split between Eurasian lynx haplotypes and *L. canadensis*) with mean of 1.18 (95% CI 0.7–1.98) Myr. Analysis of the posterior distributions of tree likelihood and other parameters using the program Tracer v.1.7.1^42^ showed ESS values > 200. TreeAnnotator v.2.6.6 was then used to remove the first 10% of trees as burnin and extract the maximum clade credibility tree with nodes scaled to the mean heights recovered by the posterior sample and visualized using FigTree v.1.4.4 (http://tree.bio.ed.ac.uk/software/figtree/).

### Population differentiation and structure

Measures of DNA polymorphism were estimated for *L. lynx* as a whole, as well as for the subspecies that we propose, i.e., *L. l. lynx* (combined West 1 and West 2 clades), *L. l. wrangeli* (East clade), and *L. l. isabellinus* (South clade). Using DnaSP v.5^38^ we calculated haplotype diversity (*h*, the probability that any two randomly sampled haplotypes are different^45^), nucleotide diversity (*π*, the average number of nucleotide differences per site), and the mean number of pairwise differences within a group (*k*).

We further estimated mismatch distributions separately for the total group and the putative subspecies (see above) to test if their frequency graph shows a chaotic/multimodal pattern characteristic for populations in demographic equilibrium, or a unimodal profile which is found in populations that experienced recent geographic expansion^46^. The test was performed using Arlequin v.3.5.2.2^47^ under the null hypothesis that the observed data fit the sudden expansion model using a generalized least-square approach with 1000 bootstrap replicates. Other statistics for analyzing population expansions or declines were also calculated using DnaSP v.5^38^, i.e., R_2_^48^ which use information on the frequency of segregating sites, and Fu’s F_s_^49^ which is a measure for estimating the amount of rare alleles (young mutations in expanding populations), where a negative value is interpreted as indicating recent demographic expansion.

In addition, a coalescent based Bayesian skyline plot^50^ was reconstructed using the BEAST v.2.6.6 program package^44^ with HKY + G and empirical base frequencies under the substitution model and 10^7^ MCMC repetitions. We used a strict molecular clock model, setting tuning parameters of operators to yield effective sample sizes (ESS) larger than 200. Convergence was visually checked in Tracer v.1.7.1^42^.

## Supplementary Information

Supplementary Information 1.

Supplementary Information 2.

Supplementary Information 3.

Supplementary Information 4.
