# Supplementary figures and images for: Phylogenetic analyses of Eurasian lynx (Lynx lynx Linnaeus, 1758) including new mitochondrial DNA sequences from Iran
Source: Sci Rep. 2022 Feb 28;12:3293. doi: 10.1038/s41598-022-07369-z (PMC8885656; doi:10.1038/s41598-022-07369-z)

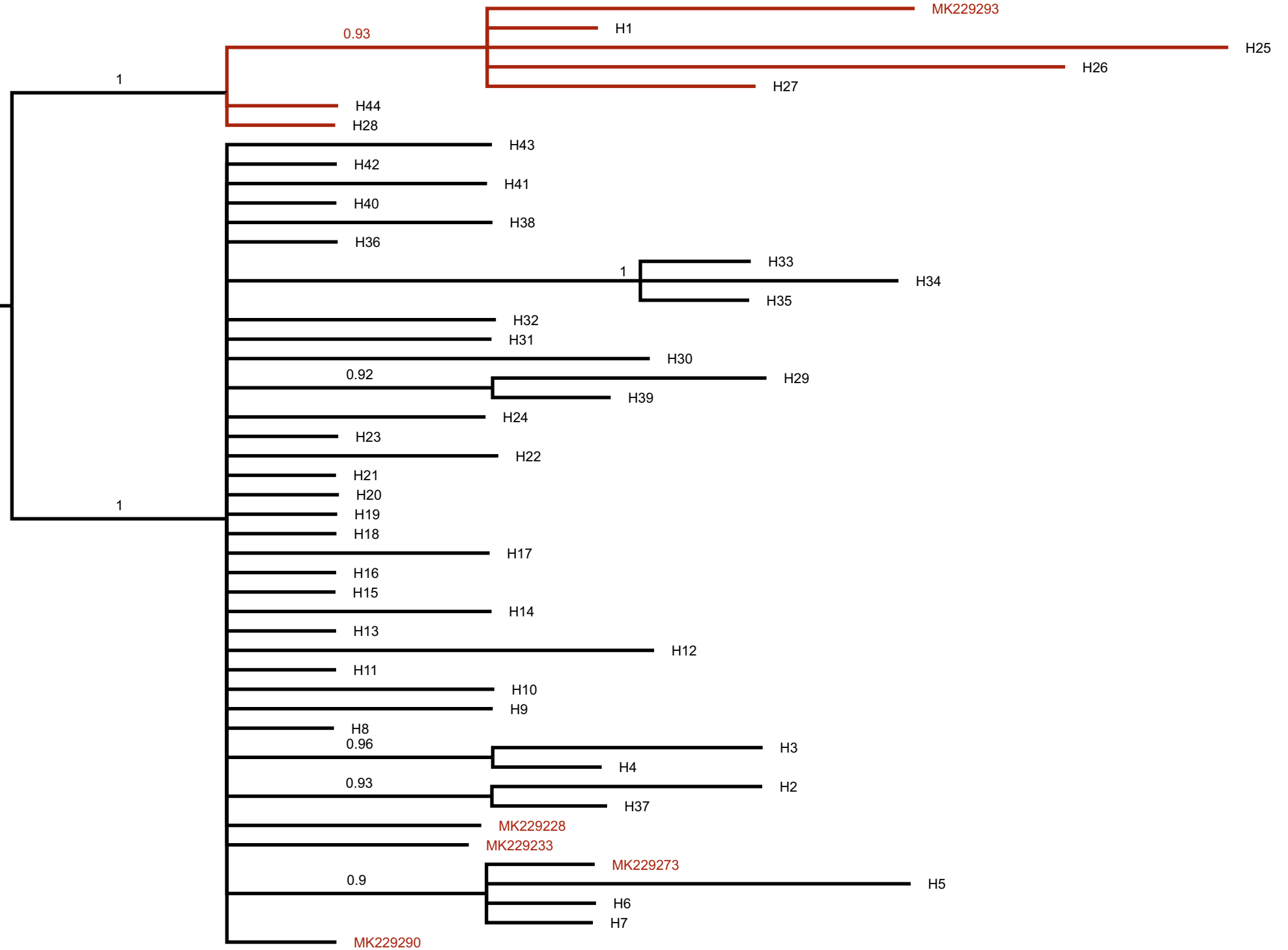

Supplement: Supplementary file 1 — Supplementary Information 1. [file 41598_2022_7369_MOESM1_ESM.pdf]

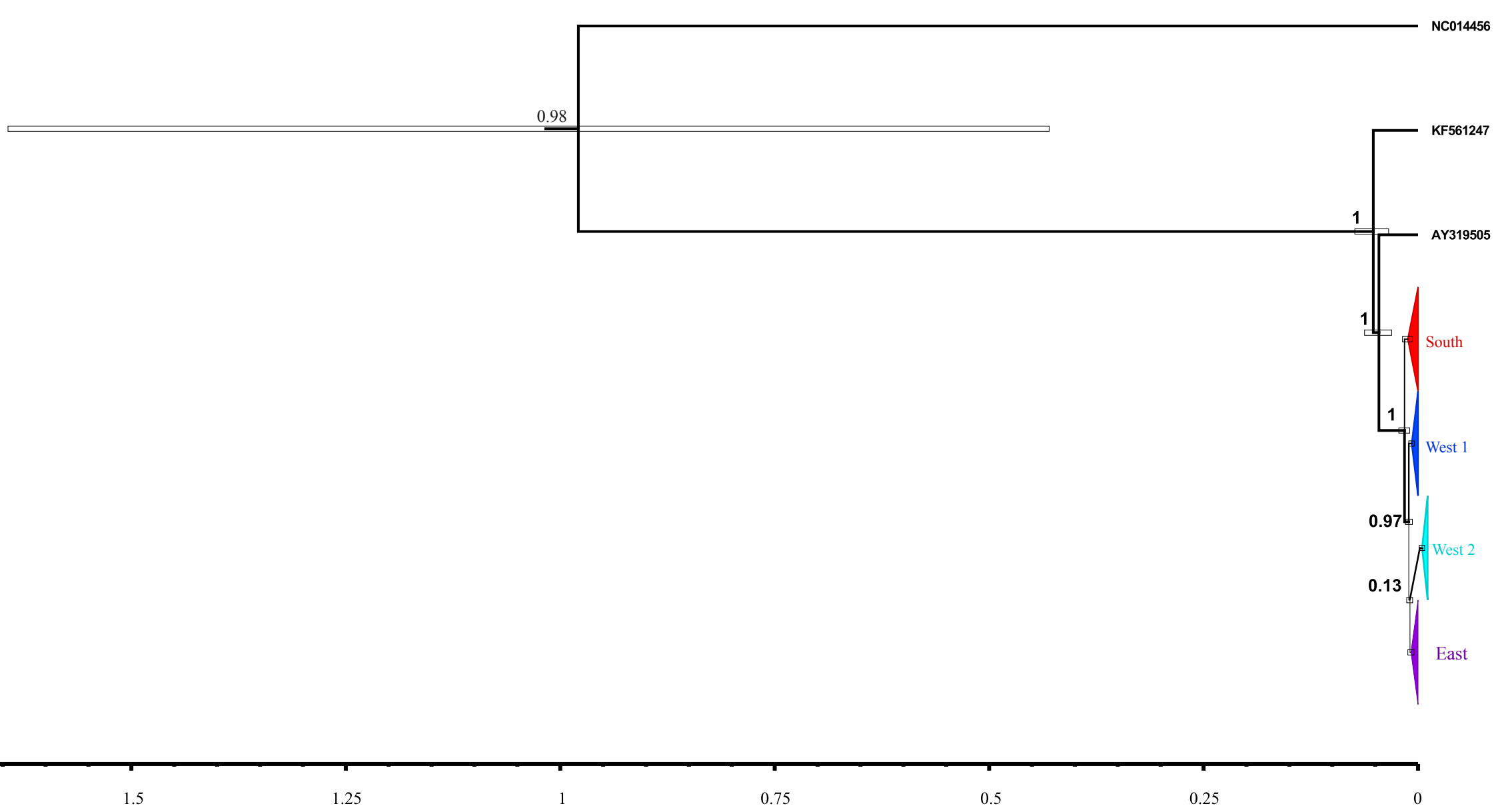

Supplement: Supplementary file 2 — Supplementary Information 2. [file 41598_2022_7369_MOESM2_ESM.pdf]
