# Supplementary material for: Phylogenetic analyses of Eurasian lynx (Lynx lynx Linnaeus, 1758) including new mitochondrial DNA sequences from Iran
Source: Sci Rep. 2022 Feb 28;12:3293. doi: 10.1038/s41598-022-07369-z (PMC8885656; doi:10.1038/s41598-022-07369-z)
Supplement: Supplementary file 4 — Supplementary Information 4. [file 41598_2022_7369_MOESM4_ESM.docx]

Figure S1. Phylogeny of Eurasian lynx based on partial mitochondrial control region sequences (326 bp).

Figure S2. Time-calibrated CR mitochondrial phylogeny of Eurasian lynx and other lynx species (*L. rufus*, *L. pardinus*, and *L. canadensis*).
